# Supplementary material for: DRAG in situ barcoding reveals an increased number of HSPCs contributing to myelopoiesis with age
Source: Nat Commun. 2023 Apr 17;14:2184. doi: 10.1038/s41467-023-37167-8 (PMC10110593; doi:10.1038/s41467-023-37167-8)
Supplement: Supplementary file 1 — Supplementary Information [file 41467_2023_37167_MOESM1_ESM.pdf]

Supplementary information, Urbanus J et al

Supplementary Methods

Single barcoded MEF clones. Embryos from DRAG1 homozygote CagCreERT2 homozygote mice were harvested at day 14.5 and PCR genotyped. Extracted MEFs were immortalized by transduction with p53shRNA (in pRetro-super backbone, kindly provided by M. v. Lohuizen) and puromycin selected. Recombination of the DRAG transgene was induced in vitro using 5 µM 4-OH-Tamoxifen. Following induction, single GFP+ cells were grown by limiting dilution. Clones were individually Sanger sequenced to identify DRAG barcodes. To confirm barcode identities, clones were also deep-sequenced using the same PCR pipeline (including capture) as used for other DRAG samples (see below). To create MEF mixes, 7 clones, each harboring a different barcode, were mixed in a ratio of 64:32:16:8:4:2:1. Resulting mixtures were used to prepare pools of 50,000, 12,500, 3,125, 781 and 195 cells, and samples were further processed as described for DRAG samples.

DRAG induction in mammary gland tissue

ROSACre$^{ERT2+/-}$ DRAG$^{+/-}$ mice were induced by one single injection of tamoxifen (0.1mg/g body weight) at P21. Mammary glands were collected 1 month after induction. Single cell dissociation was performed through enzymatic digestion (5mg/ml collagenase (Roche, 57981821) and 200U/ml hyaluronidase (Sigma, H3884) for 1h30 at 37°C under agitation. Subsequently, cells were treated with trypsin for 1 min and DNAse I and dispase for 5min at 37°C. Cell suspension was filtered through a 40µm cell strainer, and cells were stained in FACS buffer (PBS, EDTA 5mM, BSA 1%, FBS 1%) using a ‘lineage cocktail’ in APC (CD45 clone 30-F11, CD31 clone MEC13.3, Ter119+ clone TER-119, all diluted 1/100), PE EpCAM (clone G8.8, 1/100), APC/Cy7 CD49f (clone GoH3, 1/100) and DAPI. All antibodies were purchased from Biolegend. Cells were analyzed on a FACSAria™ flow cytometer (BD Biosciences), and results were analysed using FlowJo software.

DRAG induction in brain

CagGCre-ER$^{TM+/-}$ DRAG$^{+/-}$ mice aged 15 weeks for the uninduced group and 37 weeks for the induced group were sacrificed and their head fixed in 4% formaldehyde in PBS. Tamoxifen induction was performed in 17 week old mice as described in the tamoxifen induction section. Brains were sectioned into 30-um sections using a Leica vibratome, and sections were mounted on glass slides. Three sections, from the same rostro-caudal level in each mouse, were analyzed

per mouse. Sections were imaged using a Zeiss LSM 700 confocal microscope on Tile Scan mode, using a 20x objective and 3-µm optical sections. All microscope settings (e.g., laser, gain, offset, pinhole, averaging) were kept constant for each image.

Barcode Preprocessing and Filtering.

Each recombined sequence includes nucleotide additions and deletions (referred to as the 'barcode') and constant parts that flank both sides of this barcode. Moreover, each barcode was associated with a random unique molecular identifier (UMI) of 12bp during the tagging PCR step.

Preprocessing. We use the pipeline described below to demultiplex fastq files and identify the reads that match a potential recombination of the DRAG construct. First the bcl2fastq (Illumina) program is used to demultiplex the fastq files based on the i7 index sequence. Only records that match the i7 index perfectly are considered for the next step. In the constant part of the V and the J, the reads tend to be error-prone and a consensus sequence with Ns is manually created. The Xcalibr program (https://github.com/NKI-GCF/xcalibr) is then used to extract counts for all combinations of the 12bp UMI and the recombined barcodes only for the reads that contain the constant sequence of the V (ctcgaggtcatcgaagtatcaag) at the expected coordinates. After this, the J constant part (tagcaagctcgagagtagacctactggaatcagaccgccaccatggtgagc) is aligned to the barcode part using the NBCI blast2 program [1]. When a suitable match is found, the barcode is trimmed at the start coordinate of the match, resulting in the final matrix.

Barcode filtering. We used the steps described below to identify barcode sequences and remove PCR and deep-sequencing errors. First, we removed any barcode and associated UMI containing one or multiple 'N' values (within either the barcode, constant flanking parts or UMI). Second, barcodes that did not have an exact match to the expected constant parts (for the V region : cctcgaggtcatcgaagtatcaag and the J region : tagcaagctcgagagtagacctactggaatcaga) of the V and J that precede or follow the barcode were removed. Third, when multiple sequences were found associated with a single UMI, only the most frequently occurring barcode associated with that UMI was kept. Note that in theory, the number of different UMIs (4^{12}) should be in excess to the number of template molecules present in a PCR pre-amplified sample at the point at which tagging takes place (with a maximum of 5.10^4 expected molecules). However, we did observe rare cases in which the same UMI was associated with multiple true barcodes, presumably due to a lower than expected diversity of the tagging primer and a biased composition. Dominant barcodes that were associated with a UMI are highly likely to also be

the dominant barcode associated with another UMI when samples are sequenced sufficiently deep and therefore likely to be true barcodes. Fourth, we removed UMI-barcode combinations with a read count of 10 or below to remove low-abundant combinations from our main list of barcodes. As a fifth step, we summed up the read counts for all UMI associated with the set of remaining barcodes, including the UMI from UMI-barcode combinations below the 10-read threshold for which the barcode matched one of the barcodes that passed the 10-read threshold.

To verify that the barcodes obtained match the expected structure of a VDJ recombination product, we developed an algorithm to compare barcodes to the original VDJ template and identify which nucleotides were deleted due to exonuclease activity and which ones were inserted due to Tdt activity. This enabled us to recognize barcodes containing residual error, and also to quantify barcode creation patterns (i.e. the number of deleted and inserted nucleotides at the junctions between V, D and J segments per barcode). Specifically, the algorithm performed several matching steps to the following template parts: V element=TCCAGTAG, forward D element=TCTACTATCGTTACGAC, reverse D element=GTCGTAACGATAGTAGA, and J element=GTAGCTACTACCG. The locations in between the V and D, as well as the D and J elements, are the sites where recombination can occur. Note that the residues matching the V and J region were actually longer but we did not observe deletions extending beyond the above-described residues. The algorithm started by performing exact matching to the V element, comparing residues from left to right, and to the J element from right to left. This resulted in matched V and J parts and a ‘middle part’ that contains a part of the forward or reverse D element (provided that this was not completely deleted during recombination). In order to find the most likely match of the middle part to the D element, we separately searched for the longest matches to both the forward and the reverse D element, while considering that there could be residual sequencing error within this constant element. We achieved this by starting with an attempt to match to the longest possible sequence (i.e., the entire D element of length 17 nucleotides), and decreasing the attempted match length by 1 until a match could be established. In this matching process we first searched for exact matches amongst all permutations of the considered length. For example, a comparison of the remaining middle part to a part of the D element of length 15 involves comparing to three potential D parts, i.e., a D part where two nucleotides are deleted on the left side, one where one nucleotide is deleted on both sides, and one where two nucleotides are deleted on the right side. In case no exact match could be found for the considered length, we searched for potential approximate matches in which a mismatch of a single nucleotide was allowed, provided that

the mismatch did not occur in one of the two flanking residues on the left and right side (note that this implies that the minimal D fragment length for which such a mismatch can be detected is a length of 5 nucleotides). This is because a mismatch close to the flanking regions may easily be caused by nucleotide deletions and insertions during the recombination process, whereas single mismatches at locations further away from the D element flanks are more likely to be due to sequencing errors.

Having established the longest match to both the forward and the reverse D element, the longest of these two was selected for further analysis, provided that the match length was at least five nucleotides. When this longest match contained a nucleotide mismatch, the sequence was no longer considered as real and thus discarded. However, when an exact match was observed, the remaining left and right flanks of the middle part were considered insertions between V and D, and between D and J, respectively. The nucleotides of the original forward or reverse D template that were no longer present in such barcodes were considered to be deleted. For a case with a longest match of at most four nucleotides, we considered it most likely that the entire D template had been erased during recombination. In that special case, the remaining few nucleotides were assigned as follows: (i) to insertions between the V and D element in case the already recovered V element was empty and the recovered J element was not empty (because we wanted to consider the possibility that the V element was in fact non-empty but contained residual sequencing error; see below), (ii) to insertions between the D and J element in case the already recovered V and J elements were both empty (in which case we considered it likely that in fact the recovered J element was non-empty because of the large number of residues that would have been deleted on that flank otherwise), and (iii) to insertions on both insertion flanks that were equally divided amongst the V/D and D/J flank for an even number of nucleotides and with one insertion more for the D/J flank for an uneven number.

The above part of the algorithm only considered potential residual sequencing errors within the D element and not within the V and J constant elements, which was done subsequently. This was achieved by considering whether extension of the earlier detected exact matches to the V and J templates into the determined insertions between V/D and D/J, respectively, would lead to longer matches when allowing for a single mismatch in either of the constant regions. In the case of a mismatch in the constant regions, the mismatch was allowed to occur at the nucleotide immediately close to the already detected V and J parts (in the rightward and leftward direction, respectively). When a second mismatch was detected within either the second or the third

nucleotide flanking the already determined exact match, an extension was not accepted. In that case, the deletions on the right side of the original V template and on the left side of the J template were determined based on the missing nucleotides. However, if the two nucleotides (in second and third position away from the exact match) did match to the original template, the position immediately flanking the earlier detected exact match was considered as an error and in that case the sequence was discarded. In summary, the algorithm detected residual sequencing errors within the constant V, D and J elements, and it determined both the insertions between V/D and D/J and the deletions from the original V, D and J templates. Note that the algorithm detected only a limited number of spurious sequences, because most of those were already removed by the other steps applied to the cellular barcoding data.

Probability Generation Model

We used the barcode sequence lists from the previous filtering step to infer the properties of the recombination process that produce these barcodes using the IGoR algorithm, similarly to previous work [2]. To adapt IGoR to fit the DRAG system, the genomic templates for recombination were redefined as the V, J, and D genes in the DRAG construct, adding also the inverted form of the D segment. Then, IGoR was run using all unique barcode as inputs to infer the probabilities of each possible insertion (ins) and deletion (del) scenario.
The inferred probability of recombination of a barcode σ is

$$(1) P_{gen}(\sigma) = \sum_{scenario \to \sigma} P_{\mathrm{recomb}}(scenario), \quad (1)$$

i.e. the sum of the probabilities $P_{\mathrm{recomb}}(scenario)$ of all recombination scenarios leading to barcode σ. Scenario probabilities are in turn given by:

$$(2) P_{\mathrm{recomb}}(scenario) = P(D)P(delV)P(delD)P(delJ)P(insVD)P(insDJ)$$

where P(D) corresponds to the probability the usage of the D or the inverted D; P(delV), P (ins VD) and P (ins DJ) correspond to insertion between the V and D segments and D and J segments respectively; P(delV), P(delD) and P(delJ) correspond to the deletion in the V,D or J segment respectively.

P(D) is calculated from the occurrence of the inverted and non-inverted form in the data, with P(D non inverted)=0.88 and P(D inverted)= 0.12. The probabilities for the insertion (P(ins))

depend both on the length of the segment (lenVD) and on its composition through a Markov Model:

$$(3)P(insVD) = P(lenVD)P_{VD}(\sigma_1)\prod_{i>2} P_{VD}(\sigma_i|\sigma_{i-1})$$

where the product runs over the non-templated inserted nucleotides. $P(insDJ)$ is defined similarly. The inferred parameters are summarized in the Table S7-9. In the Markov model, the insertions are parameterized both by their length, and by the probability of insertion of each of the four bases, given what was the last insertion.

Note that deletion numbers for the V and J segments include the possibility of short palindromic insertions, which are given by negative deletions. Negative deletion means that, instead of being deleted, the sequence gets up to 4 additional nucleotides that are reverse-complement to the last ones. Since the J segment is longer, it can have more deletions. The D segment can be deleted from both sides, and these are correlated, so the model incorporates a joint deletions distribution, different for the reverse D. The inferred probabilities for P(del) are summarized in the Table S4-6.

This inferred model was used to calculate a generation probability (P_{gen}) for every barcode using Eq. (1). The generation probability of each barcode determines how probable it is to find two cells with the same barcode, coming from different recombination events. Where indicated, we discarded barcodes above a certain threshold generation probability (P_{gen}) to eliminate barcodes that are likely to be independently generated in more than one cell.

Barcode Analysis.

All analyses were carried out using R software (v3-v4) [3]. After running the barcode filtering pipeline, the data was placed in a count matrix for each barcode in rows and samples in columns. All barcodes that had a read value below 0.003% of total reads were set to zero to clean residual errors. The reads per sample were then renormalized to 1 or to cell numbers obtained from sorting. This renormalized matrix was used for diversity analysis. The chao index was computed using a custom script on the renormalized read to cell numbers per duplicate for each sample, using the formula below:

$$(4)chao2 = N_{obs} + \frac{1}{2} \times \frac{N_1^2}{2N_2}$$

where N_{obs} is the number of barcodes observed in both duplicates, N_1 the number of barcodes present in one duplicate and N_2 the number of barcodes shared between duplicates. As there are different ways to compute diversity from occurrence data, we compare the results for different indexes: the bias-corrected chao2 (chao2corr), the first order jackknife (jack1), and the bootstrap (boot) (Fig. S4B) using the vegan package [4]. The formulas for computing theses indexes are below:

$$(5) chao2corr = N_{obs} + \frac{1}{2} \times \frac{N_1 \times (N_1 - 1)}{2(N_2 + 1)}$$

$$(6) jack1 = N_{obs} + \frac{1}{2} \times N_1$$

$$(7) boot = N_{obs} + \sum (1 - p_i)^2$$

where N_{obs}, N_1 and N_2 are defined as before and p_i is the frequency of the barcodes merging both duplicates.

The absolute number of HSC per blood sample was extrapolated using the chao2 index and the percentage of GFP+ cells in the sort sample (as all the bone marrow sample was sorted). We estimated that between 100-200µl of blood was collected at each time point. For the evolution of HSC diversity over time, Renyi indices were computed using a custom script on renormalized read to cell numbers to 1 per duplicate for each sample, using the formula below [5]:

$$(8) {}^{q}D = \left(\sum_{i}^{s} p_i^q \right)^{\frac{1}{1-q}}$$

where q is the order of the diversity index, p_i is the frequency of a given barcode in the sample. q=0 is the richness in the sample, the number of barcodes present, q=1 is the Shannon index, q=2 is the Simpson index. For the Shannon index q=1, the limit of the ${}^{q}D$ formula gives:

$$(9) {}^{1}D = \exp \left(\sum_{i}^{s} p_i \ln p_i \right)$$

Renyi indexes were then analyzed using a gamma generalized linear mixed model, as described below.

For heatmap analyses (Fig. 3B, 4B, S4A), barcodes not present in both duplicates were removed, the technical duplicates were summed, renormalized to the arbitrary value of 10^5 for

visualization, and transformed using the hyperbolic arcsine function. Where applicable, barcodes with a $P_{gen}>10^x$ were filtered out. Heatmaps were generated using the heatmap package gplots [6], using Euclidean distance and complete linkage.

To classify barcodes into LSK, MP, and M categories (Fig. 3C), we used a previously described hand tailored classifier [7,8]. In summary, barcodes were classified into categories based on their presence or absence in the given cell type (LSK, MP, or M). The contribution of the sum of all barcodes in each category was computed and is displayed in Fig. 3C.

For the analysis of barcode sharing between duplicates, time points and mice, the Jaccard index was computed using the biomod2 [9] and the ade4 [10] packages, transformed into a fraction of barcode shared (1-jaccard^2) and then plotted using the corrplot package [11].

FACS data were analyzed using FlowJo software (*Becton Dickinson).*

Gamma generalized linear mixed model for diversity over time

Since the Renyi entropy indices are positive and continuous variables, gamma generalized linear mixed models were fitted to the data. Let Y_{ijk} be a random variable representing the richness measured on mouse i, month j, and subsample k. The conditional distribution $Y_{ijk}|m_i, s_{ij} \sim \text{Gamma}(\mu_{ijk}, \phi)$ was assumed, with $m_i \sim N(0, \sigma_m^2)$ random mouse effects and $s_{ij} \sim N(0, \sigma_s^2)$ random sample effects, and ϕ the dispersion parameter. These random effects were included to model the correlation between the observations taken on the same mouse and duplicates. The mean was modelled with an identity link, and a piecewise-linear predictor over time (months) was used, i.e.

$$(10)\mu_{ijk} = m_i + s_{ij} + \beta_0 + \beta_1 * \text{month}_\text{j} + \beta_2 * (\text{month}_\text{j} - \kappa) * \text{I}(\text{month}_\text{j} > \kappa)\ (1),$$

where κ is the break point estimated by maximising the profile log-likelihood of the model, and $\text{I}(\text{month}_\text{j} > \kappa)$ is a dummy variable assuming value 1 when $\text{month}_\text{j} > \kappa$ and 0 otherwise. Maximum likelihood estimates were obtained using the Laplace approximation for the integrals in the log-likelihood function. The best fit parameter estimates are summarized in Table S10. Goodness-of-fit of the models were assessed using half-normal plots with simulation envelopes [12]. The models were fitted using package lme4 [13] from the R software.

Gamma generalized linear mixed model for cell output per barcode over time

The cell output data consisted of continuous, strictly positive data, and therefore gamma generalized linear mixed models were used for this analysis, including random intercepts and slopes over time per mouse, and different dispersion per mouse. Let Y_{ijk} be the response for the

i-th mouse, j-th tag and k-th time point. It was assumed that $Y_{ijk}|m_{0i}, m_{1i}, t_{0ij} \sim \text{Gamma}(\mu_{ijk}, \phi_i)$, with $m_{0i} \sim N(0, \sigma_{m0}^2)$ and $m_{1i} \sim N(0, \sigma_{m1}^2)$ the random intercepts and slopes per mouse, respectively, $t_{0ij} \sim N(0, \sigma_{t0}^2)$ the random intercept for tag j within mouse i, and ϕ_i the mouse-specific dispersion parameter. These random effects were included to model the correlation between the observations taken on the same mouse and tag. The mean was modelled with a log-link, such that

$$(11)\ \log(\mu_{ijk}) = \beta_0 + m_{0i} + t_{0ij} + (\beta_1 + m_{1i}) * \text{month}_k\ (2),$$

and the dispersion was also modelled using a log-link, and included different intercepts per mouse, i.e.

$$(12)\ \log(\phi_i) = \gamma_i\ (3)$$

Maximum likelihood estimates were obtained using the Laplace approximation for the integrals in the log-likelihood function. Two statistical hypotheses were tested: (1) $H_0: \beta_1 = 0$ versus the alternative $H_a: \beta_1 \neq 0$, which is equivalent to testing whether there was a trend over time, and (2) $H_0: \sigma_{t0}^2 = 0$ versus the alternative $H_a: \sigma_{t0}^2 > 0$, which is equivalent to testing whether within a mouse at a given time point all barcodes are equivalent. Hypotheses were tested using likelihood-ratio tests for nested models. Goodness-of-fit of the models were assessed using half-normal plots with simulation envelopes [12]. The models were fitted using package glmmTMB [14] for R software.

Bone marrow single cell transcriptomics for the HSPC composition of Figure 2.

Bone marrow cell preparation: At sacrifice, BM was harvested from femurs, tibias and ilia and enriched using anti-CD117 magnetic beads (Miltenyi). The c-kit$^+$ fraction was stained with antibodies against CD117 (c-kit APC, clone 2B8, Biolegend) and Sca-1 (Pacific Blue, clone D7, eBioscience). Cell sorting was performed on a FACSAria™ (BD Biosciences) using a 70 µm nozzle at precision 0/16/0 and high efficiency. LSK (c-Kit$^+$sca1$^+$) cells were sorted into GFP$^+$ and GFP$^-$ cells from the c-Kit-enriched bone marrow fraction. <u>10X Genomics V2 3' Library preparation:</u> The two sorted fractions (3,000 GFP$^+$ and GFP$^-$ cells) were then processed using the V.2 10X genomics protocol. cDNA amplification was performed with 11-13 PCR cycles depending on the targeted cell recovery, as per the manufacturer's recommendations. Sequencing was performed on a NovaSeq (illumina) on paired-end (PE28-8-91). <u>Single-Cell RNA-seq analysis:</u> Sequencing reads were processed using the default cell-ranger pre-processing pipeline and were aligned to the mouse mm10 reference genome. Gene-expression count matrices for the 642 GFP$^+$ cells and 2231 GFP$^-$ cells were loaded into R and analysed

using Seurat v4.0. We performed QC by visual inspection of library sizes, numbers of genes expressed and mitochondrial content per cell. Cells with less than 500 genes or with a high percentage (> 10% of mitochondrial genes) were removed from downstream analyses. Cells with numbers of genes recovered were considered as doublets and filtered from the data. In this filter 1500 genes was used as an upper limit and this threshold was defined based on outlier points from plotting UMI counts and numbers of genes detected. After filtering, our count matrix contained 2,775 cells and 13,183 genes. Data was then normalised using scTransform [15] for which the normalized values are Pearson residuals from regularized negative binomial regression and cellular sequencing depth is used as a covariate. In our data, we observe a batch effect between GFP^+ and GFP^- LSKs, likely arising because of the parallel processing of the two samples on the 10X machine as genes showed a linear increase in expression in the GFP^+ fraction compare to the GFP^- fraction but not the inverse (Fig. S3A). To correct for this batch effect, we modelled the batch effect using a negative binomial model, with model residuals representing the batch-corrected expression values. We then performed dimensionality reduction on the 2,500 variably expressed genes using first principal component analysis followed by the non-linear dimensionality reduction technique UMAP [16] (Fig. S3B). We then performed unsupervised Louvain clustering of the data across a range of resolution parameters and chose the resolution value that led to the most stable clustering profiles [17] (Fig. S3C). This approach yielded 9 distinct clusters, which were manually annotated (LT-HSCs, MPP2-5) using existing markers and transcriptomic signatures [18,19] (Fig. 2B and S3D). Consistent with reports that HSPCs do not form discrete cell subsets [20–23] we observed that many clusters co-expressed signatures of the MPP1-5 subtypes (Figure 5C, S7A-B). In cases in which clusters could not be assigned to a single HSPC subset, we named the cluster according to the combination of the different HSC and MPP signatures that they expressed [24]. To test for possible enrichment of GFP+ cells within a given cluster, Fisher’s exact test was used. Signature expression scores were calculated using the AddModuleScore() method of Seurat V4.

Bone marrow single cell transcriptomics for Figure 5.

Bone marrow cell preparation: At sacrifice, BM was harvested from femurs, tibias and ilia and enriched using anti-CD117 magnetic beads (Miltenyi). The c-kit^+ fraction was stained with antibodies against CD117 (c-kit APC, clone 2B8, Biolegend) and Sca-1 (Pacific Blue, clone D7, eBioscience). Flow cytometry was performed on a FACSAria™ (BD Biosciences) or sh800 (Sony). DRAG barcoded LSK (c-Kit^+ $Sca1^+$ GFP^+) cells were sorted using a 70 µm nozzle at precision 0/16/0 and high efficiency. <u>10X Genomics V3 3’ Library preparation:</u> Samples were

processed using the 10X genomics Chromium Single Cell 3′ v3 kit. Specifically, 1,000-16,000 cells were loaded for each experiment for a targeted recovery of 500-10,000 cells. cDNA amplification was performed with 11-13 PCR cycles depending on the targeted cell recovery, as per the manufacturer's recommendations. Sequencing was performed on a NovaSeq (illumina) on paired-end (PE28-8-91). Single-Cell RNA-seq analysis: Raw sequencing reads were processed using Cellranger and reads were mapped to the mouse mm10 reference genome. During filtering, Gm, Rik, and Rp genes were filtered from the dataset. Cells with less than 500 genes per cell or with a high percentage (> 15% of mitochondrial genes) were removed from downstream analyses. Cells with a UMI count greater than 50,000 were considered as doublets and removed from the data. Following these filtering procedures, the average UMI count per cell was 11,829. The median number of genes detected per cell was 2,845, 2.9% mapped to mitochondrial genes. Cell cycle annotation using the cyclone method from the scran R package showed that 13,866 cells were in G1 phase, 2,230 cells were in G2M phase, and 682 cells were in S phase. Data normalization and integration were performed using the default Seurat v4 approach FindIntegrationAnchors() followed by IntegrateData(), and differentially expressed genes were determined using a logistic regression in Seurat on the non-integrated data using the FindMarkers() function. Pathway based analyses were performed using the enrichR package [25,26]. To create the aged HSPC signature we performed differential gene expression analysis between HSPCs from 6.5 month old and 19 month old mice. Genes upregulated in 19 month old mice were then aggregated into a signature using the AddModuleScore() method of Seurat. Annotation of the data was obtained by unsupervised clustering of the data followed by supervised annotation in which we mapped published signatures using the AddModuleScore() method of Seurat. The MolO LT-HSC signature was taken from [27], and the MPP2/3/4/5 signatures were taken from [19] and from [24]. Similarly to the analysis for Fig. 2, in cases in which clusters could not be assigned to a single HSPC subset, we named the cluster according to the combination of different HSC and MPP signatures that they expressed. Pseudotime analysis was performed using the destiny R package. In this diffusion map approach, the algorithm creates a pairwise cell transition probability matrix. This probability is calculated by modelling cell state transitions as a random walk, in which cells can move within a local neighbourhood specified by the parameter σ. The greater the overlap between the gene expression neighbourhoods of two cells, the higher the transition probability. Label transfer was performed in Seurat using the FindTransferAnchors() and TransferData() methods. Briefly, this approach involves projecting the PCA structure of the reference dataset onto the query dataset. Within this shared PCA projection paired mutual nearest neighbours (anchors) are defined for each

dataset. To perform label transfer, a weight matrix is defined that defines the association between query cells and anchor cells. This matrix is then multiplied by a binary classification matrix to compute a prediction score that a query cell belongs to a certain class of reference cells. In this binary classification matrix rows correspond to the different cell classes and columns correspond to the anchors. If the reference cell in the anchor pair belongs to a certain class the matrix entry is filled with a 1, otherwise 0.

Oligo sequences

Mef clones 2nd round PCR for Sanger sequencing

M1seqv2for:

ACACTCTTTCCCTACACGACGCTCTTCCGATCTNNNNCTCGAGGTCATCGAAGTA TCAAG

SM2index v2**:** GTGACTGGAGTTCAGACGTGTGCTCTTCCGATC CGTCCAGCTCGACCAGGAT

Capture

Biotinylated capture forward:

5'-BiotinTeg-CCGCTAGCGGCCAGGGCGGCCGGAGAATTGTAATACGACTCACTAT AGGGAGACGCGTGTTACCTCCTCGAGGTCATCGAAGTATCAAG

Biotinylated capture:

5'-BiotinTeg-CTATAGCGGCCGCCTAGGCCGCTCTTCAACTACC TTGTACAGCTCGTCCATGCCGAGAGTGATCCCGGCGGCGGTCACGAACTCCAGCA GGACCATGTGA

Tagging PCR

Preamp forward: ACTCACTATAGGGAGACGCGTGTTACC

Preamp reverse: GACACGCTGAACTTGTGGCCGTTTA

M1 tag forward:

ACACTCTTTCCCTACACGACGCTCTTCCGATCNNNNNNNNNNNNCCTCGAGGTCA TCGAAGTATCAAG

Illumina forward seq (Read 1): ACACTCTTTCCCTACACGACGCTCTTCCGA*T*C (*= Phosphorothioate bond)

M1 rev Read2: AGTTCAGACGTGTGCTCTTCCGATC CAGCTCGACCAGGATG*G*G

P5 forward :
AATGATACGGCGACCACCGAGATCTACACTCTTTCCCTACACGACGCTCTTCCGA
TC
P7 index rev:
CAAGCAGAAGACGGCATACGAGAT**XXXXXXXX**GTGACTGGAGTTCAGACGTGTGC
TCTTCCGATC
Complete list of the i7 indexes in Supplementary Data 1.
All oligos were ordered at IDT with HPLC purified grade.

Flow cytometry analysis and statistical testing:

Data analysis was performed using FlowJo^{TM} v.10 (TreeStar). Data was then exported from FlowJo and imported in GraphPad Prism. Where indicated, a Mann-Whitney test was performed.

Code and data availability: All data and code are available at https://github.com/TeamPerie/UrbanusCosgrove-et-al-DRAG-mouse.git

Supplementary Tables

Table S1: DRAG barcode sequences observed in MEF clone panel. The DRAG barcodes were split to see which parts of the sequence match the original V, D and J segments (template sequence on the first line) and compute the number of insertions and deletions.

MEF clone number	DRAG barcode	V	insertion between V and D	D	insertion between D and J	J	deletion V	deletion D left part	deletion D right part	deletion J
	template	TCCAGTAG		TCTACTATCGTTACGAC		GTAGCTACTACCGTAG				
3	TCCAGTATCGTTACGCTACTACCG	TCCAGTA		TCGTTAC		GCTACTACCG	G	TCTACTA	GAC	GTA
6	TCCAGTACTATCGTACTACCG	TCCAGTA		CTATCG		TACTACCG	G	TCTA	TTACGAC	GTAGC
12	TCCAGTACTATCGTTACGTAGCTACTACCG	TCCAGTA		CTATCGTTAC		GTAGCTACTACCG	G	TCTA	GAC	
13	TCCACTACTATCGTGTAGCTACTACCG	TCCA		CTACTATCGT		GTAGCTACTACCG	GTAG	T	TACGAC	
14	TCCAGTCTACTATCGTTACGACAGCTACTACCG	TCCAGT		CTACTATCGTTACGAC		AGCTACTACCG	AG	T		GT
17	TCCATCGTAGCTACTACCG	TCCA	T		C	GTAGCTACTACCG	GTAG	TCTACTAT	CGTTACGAC	
20	TCCAGTACTATCGTTACTACTACCG	TCCAGTA		CTATCGTTA		CTACTACCG	G	TCTA	CGAC	GTAG
29	TCCAGTTACGACGCTACTACCG	TCCAGT		TACGAC		GCTACTACCG	AG	TCTACTATCG	GTA	fwd
30	TCCAGTACTGTAGCTACTACCG	TCCAGTA	C		T	GTAGCTACTACCG	G	TCTACTAT	CGTTACGAC	
31	TCCAGATCTACTATCGTTACGACGCTACTACCG	TCCAG	A	TCTACTATCGTTACGAC		GCTACTACCG	TAG			GTA
33	TCCAGTATCGTTACGACGCTACTACCG	TCCAGTA		TCGTTACGAC		GCTACTACCG	G	TCTACTA		GTA
34	TCCAATCTACTATCGTTACGACGCTACTACCG	TCCA	A	TCTACTATCGTTACGAC		GCTACTACCG	GTAG			GTA
35	TCCATATCGTTAGCTACTACCG	TCCA		TATCGT		TAGCTACTACCG	GTAG	TCTAC	TACGAC	G
36	TCCAGTTCTACTATCGTTACGTAGCTACTACCG	TCCAGT		TCTACTATCGTTAC		GTAGCTACTACCG	AG		GAC	
37	TCCAACTATCGTTACGACAGCTACTACCG	TCCA		ACTATCGTTACGAC		AGCTACTACCG	GTAG	TCT		GT
38	TCCAGTCTACTATCGTTACGACGTAGCTACTACCG	TCCAGT		CTACTATCGTTACGAC		GTAGCTACTACCG	AG	T		
46	TCCAGTTCTACTATCGTTACGAGCTACTACCG	TCCAGT		TCTACTATCGTTACG		AGCTACTACCG	AG		AC	GT
48	TCCAGTACTATCGTTACGACGCTACTACCG	TCCAGTA		CTATCGTTACGAC		GCTACTACCG	G	TCTA		
53	TCCAGTTCTACTATCGTAGCTACTACCG	TCCAGT		TCTACTATC		GTAGCTACTACCG	AG		GTTACGAC	
58	TCCAGACTATCGTTAGCTACTACCG	TCCAG		ACTATCGT		TAGCTACTACCG	TAG	TCT	TACGAC	G
59	TCCGATCTACTATCGTTACGACGTAGCTACTACCG	TCC	GA	TCTACTATCGTTACGAC		GTAGCTACTACCG	AGTAG			
60	TCCAGTTACTATCGTACGTAGCTACTACCG	TCCAGT		TACTATCGT	AC	GTAGCTACTACCG	AG	TC	TACGAC	

Table S2: Stability of DRAG barcodes in MEF clones cultured up to 138 days. 7 MEF clones were cultured after being verified as being monoclonal and sequenced by either Sanger or Illumina NGS at the indicated time points. All clones showed the same sequence over time (indicated using a green tick).

		Culturing period					
MEF clone number	DRAG barcode	Day 35 sanger	Day 66 sanger deepseq	Day 80 sanger	Day 97 sanger	Day 120 deepseq	Day 138 deepseq
3	TCCAGTATCGTTACGCTACTACCG	✅	✅	✅	✅	✅	✅
6	TCCAGTACTATCGTACTACCG	✅	✅	✅	✅	✅	✅
14	TCCAGTCTACTATCGTTACGACAGCTACTACCG	✅	✅	✅	✅	✅	✅
17	TCCATCGTAGCTACTACCG	✅	✅	✅	✅	✅	✅
30	TCCAGTACTGTAGCTACTACCG	✅	✅	✅	✅	✅	✅
46	TCCAGTTCTACTATCGTTACGAGCTACTACCG	✅	✅	✅	✅	✅	✅
53	TCCAGTTCTACTATCGTAGCTACTACCG	✅	✅	✅	✅	✅	✅

Table S3: Preamp PCR cycles

cells/half-sample	#preamp cycles
300,000-600,000	6
150,000-300,000	7
75,000-150,000	8
35,000-75,000	9
17,500-35,000	10
9,250-17,500	11
4,600-9,250	12
2,300-4,600	13
1,150-2,300	14
575-1,150	15
300-575	16
150-300	17
75-150	18
36-75	19
18-36	20
9-18	21

Table S4: Inferred probabilities of V and J segment deletions.

**Number of deletions**	*P(delV)*	*P(delJ)*
**-4**	0.00023	0.00373
**-3**	0.00027	0.00675
**-2**	0.00158	0.04580
**-1**	0.00143	0.09663
**0**	0.06288	0.14633
**1**	0.04412	0.05708
**2**	0.42011	0.08510
**3**	0.11279	0.21527
**4**	0.19543	0.09233
**5**	0.12640	0.04405
**6**	0.02430	0.03664
**7**	0.01048	0.02933
**8**	6.79E-20	0.03713
**9**	0	0.0232225
**10**	0	0.0259772
**11**	0	0.0245276
**12**	0	0.00795459
**13**	0	0.00121003
**14**	0	0.00381861
**15**	0	0.00561702
**16**	0	0.00199825
**17**	0	0.00021349
**18**	0	0.00929067

Table S5: Inferred joint probabilities for D segment deletions. 5' deletions are depicted in rows, 3' deletions in columns

P(delD)	**0**	**1**	**2**	**3**	**4**	**5**	**6**	**7**	**8**	**9**	**10**	**11**	**12**	**13**	**14**	**15**	**16**
**0**	0.012	0.005	0.009	0.005	0.011	0.003	0.009	0.009	0.003	0.001	0.000	0.000	0.001	0.000	0.000	0.000	0.000
**1**	0.031	0.011	0.020	0.020	0.030	0.009	0.012	0.020	0.005	0.001	0.002	0.003	0.004	0.001	0.002	0.005	0.016
**2**	0.009	0.002	0.003	0.003	0.004	0.002	0.002	0.002	0.000	0.000	0.000	0.002	0.001	0.000	0.004	0.008	0.000
**3**	0.032	0.015	0.019	0.019	0.031	0.009	0.017	0.024	0.007	0.002	0.002	0.002	0.002	0.000	0.002	0.000	0.000
**4**	0.021	0.009	0.011	0.009	0.011	0.005	0.008	0.007	0.003	0.001	0.001	0.002	0.001	0.003	0.000	0.000	0.000
**5**	0.016	0.003	0.007	0.005	0.006	0.001	0.002	0.002	0.001	0.000	0.000	0.000	0.000	0.000	0.000	0.000	0.000
**6**	0.019	0.004	0.007	0.006	0.007	0.004	0.005	0.003	0.000	0.001	0.000	0.001	0.000	0.000	0.000	0.000	0.000
**7**	0.003	0.001	0.002	0.001	0.001	0.000	0.000	0.000	0.004	0.000	0.000	0.000	0.000	0.000	0.000	0.000	0.000
**8**	0.020	0.004	0.008	0.009	0.012	0.003	0.001	0.002	0.001	0.000	0.000	0.000	0.000	0.000	0.000	0.000	0.000
**9**	0.003	0.001	0.001	0.000	0.000	0.000	0.000	0.000	0.000	0.000	0.000	0.000	0.000	0.000	0.000	0.000	0.000
**10**	0.001	0.000	0.001	0.000	0.000	0.001	0.000	0.000	0.000	0.000	0.000	0.000	0.000	0.000	0.000	0.000	0.000
**11**	0.002	0.002	0.002	0.000	0.000	0.000	0.000	0.000	0.000	0.000	0.000	0.000	0.000	0.000	0.000	0.000	0.000
**12**	0.002	0.001	0.000	0.002	0.000	0.000	0.000	0.000	0.000	0.000	0.000	0.000	0.000	0.000	0.000	0.000	0.000
**13**	0.001	0.001	0.001	0.000	0.000	0.000	0.000	0.000	0.000	0.000	0.000	0.000	0.000	0.000	0.000	0.000	0.000
**14**	0.001	0.000	0.000	0.000	0.000	0.000	0.000	0.000	0.000	0.000	0.000	0.000	0.000	0.000	0.000	0.000	0.000
**15**	0.001	0.000	0.000	0.000	0.000	0.000	0.000	0.000	0.000	0.000	0.000	0.000	0.000	0.000	0.000	0.000	0.000
**16**	0.000	0.000	0.000	0.000	0.000	0.000	0.000	0.000	0.000	0.000	0.000	0.000	0.000	0.000	0.000	0.000	0.000

Table S6: Inferred probabilities for inverted D segment deletions. 5’ deletions are depicted in rows, 3’ deletion in columns

P(delD)	**0**	**1**	**2**	**3**	**4**	**5**	**6**	**7**	**8**	**9**	**10**	**11**	**12**	**13**	**14**	**15**	**16**
**0**	0.012	0.006	0.004	0.007	0.000	0.046	0.009	0.003	0.015	0.004	0.001	0.000	0.000	0.000	0.000	0.000	0.000
**1**	0.000	0.000	0.000	0.000	0.000	0.002	0.000	0.000	0.000	0.000	0.000	0.009	0.000	0.001	0.025	0.015	0.003
**2**	0.020	0.009	0.015	0.013	0.012	0.128	0.026	0.017	0.034	0.010	0.002	0.007	0.001	0.011	0.009	0.008	0.000
**3**	0.005	0.021	0.003	0.001	0.001	0.006	0.001	0.001	0.005	0.000	0.000	0.000	0.000	0.000	0.030	0.000	0.000
**4**	0.003	0.002	0.001	0.001	0.001	0.007	0.000	0.000	0.000	0.000	0.002	0.002	0.000	0.000	0.000	0.000	0.000
**5**	0.000	0.014	0.011	0.009	0.005	0.021	0.003	0.000	0.004	0.002	0.039	0.000	0.000	0.000	0.000	0.000	0.000
**6**	0.001	0.004	0.003	0.004	0.002	0.003	0.000	0.004	0.002	0.014	0.000	0.000	0.000	0.000	0.000	0.000	0.000
**7**	0.006	0.002	0.004	0.003	0.003	0.012	0.001	0.004	0.012	0.010	0.000	0.000	0.000	0.000	0.000	0.000	0.000
**8**	0.001	0.001	0.000	0.000	0.000	0.000	0.000	0.001	0.001	0.000	0.000	0.000	0.000	0.000	0.000	0.000	0.000
**9**	0.000	0.000	0.000	0.000	0.001	0.000	0.008	0.000	0.000	0.000	0.000	0.000	0.000	0.000	0.000	0.000	0.000
**10**	0.000	0.000	0.000	0.000	0.000	0.002	0.000	0.000	0.000	0.000	0.000	0.000	0.000	0.000	0.000	0.000	0.000
**11**	0.000	0.000	0.001	0.004	0.006	0.000	0.000	0.000	0.000	0.000	0.000	0.000	0.000	0.000	0.000	0.000	0.000
**12**	0.000	0.000	0.000	0.000	0.000	0.000	0.000	0.000	0.000	0.000	0.000	0.000	0.000	0.000	0.000	0.000	0.000
**13**	0.000	0.000	0.002	0.000	0.000	0.000	0.000	0.000	0.000	0.000	0.000	0.000	0.000	0.000	0.000	0.000	0.000
**14**	0.002	0.006	0.000	0.000	0.000	0.000	0.000	0.000	0.000	0.000	0.000	0.000	0.000	0.000	0.000	0.000	0.000
**15**	0.001	0.000	0.000	0.000	0.000	0.000	0.000	0.000	0.000	0.000	0.000	0.000	0.000	0.000	0.000	0.000	0.000
**16**	0.000	0.000	0.000	0.000	0.000	0.000	0.000	0.000	0.000	0.000	0.000	0.000	0.000	0.000	0.000	0.000	0.000

Table S7: Inferred parameters of the Markov model for insertions between V and D segments, with the probabilities for inserting different bases n1 in rows, given the last inserted base n2 in columns, in the 5’ direction.

$P_{VD}(n_1\|n_2)$	**A**	**C**	**G**	**T**
**A**	0.20	0.10	0.24	0.26
**C**	0.26	0.51	0.16	0.39
**G**	0.41	0.13	0.49	0.16
**T**	0.131	0.25	0.10	0.18

Table S8: Inferred parameters of the Markov model for insertions between the D and J segments, with the probabilities for inserting different bases n1 in rows, given the last inserted base n2 in columns, in the 3’ direction.

$P_{DJ}(n_1\|n_2)$	**A**	**C**	**G**	**T**
**A**	0.26	0.18	0.24	0.09
**C**	0.17	0.46	0.14	0.41
**G**	0.39	0.16	0.53	0.29
**T**	0.17	0.20	0.09	0.20

Table S9: Inferred probabilities for insertions

number of insertions	*P(insVD)*	*P(insDJ)*
0	0.77	0.38
1	0.05	0.09
2	0.05	0.14
3	0.04	0.16
4	0.04	0.10
5	0.02	0.05
6	0.02	0.04
7	0.01	0.02
8	0.01	0.01
9	1E-03	0.01
10	3E-03	0.01
11	1.03E-03	1.80 E-03
12	3.87E-04	2.72 E-03
13	2.82E-04	3.94 E-04
14	0	1.03 E-03
15	5.49E-05	0
16	0	0
17	4.76E-05	0
18	2.46E-26	2.24E-04
19	0	1.27E-10

Table S10: Parameter estimates from equation (1) of the gamma generalized linear mixed model for barcode diversity over time for different Renyi indexes.

Renyi index	$\hat{\kappa}$	$\widehat{\beta_0}$	$\widehat{\beta_1}$	$\widehat{\beta_2}$
richness	7.02	63.27	-7.55	10.67
shannon	7.02	53.04	-6.32	8.77
simpson	7.02	45.79	-5.44	7.33
hill3	7.01	40.54	-4.8	6.32
hill4	7.01	36.85	-4.33	5.63
hill5	7.02	34.24	-4.00	5.17
hill6	7.02	32.35	-3.76	4.84

Supplementary Figures:

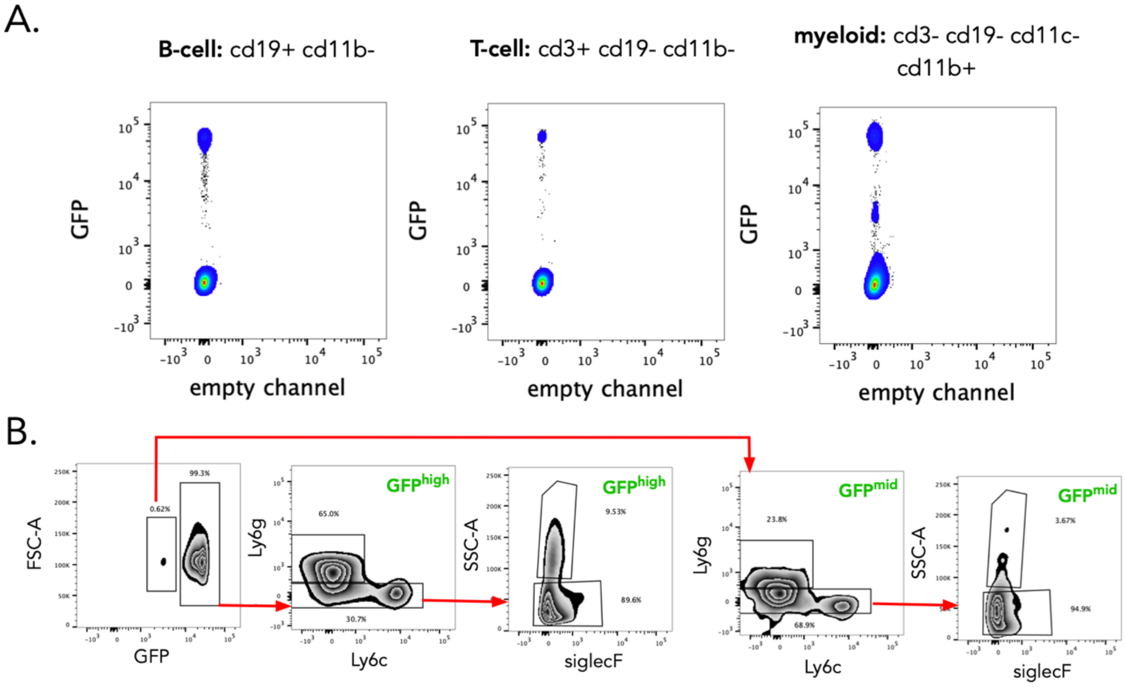

Figure S1: Analysis of GFP expression levels upon DRAG recombination in different cell compartments. (A) DRAG induced GFP expression within lymphoid and myeloid lineages. Only within the myeloid lineage a separate GFP^{mid} population is observed. (B) surface marker expression patterns for GFP^{mid} and GFP^{high} myeloid cell populations.

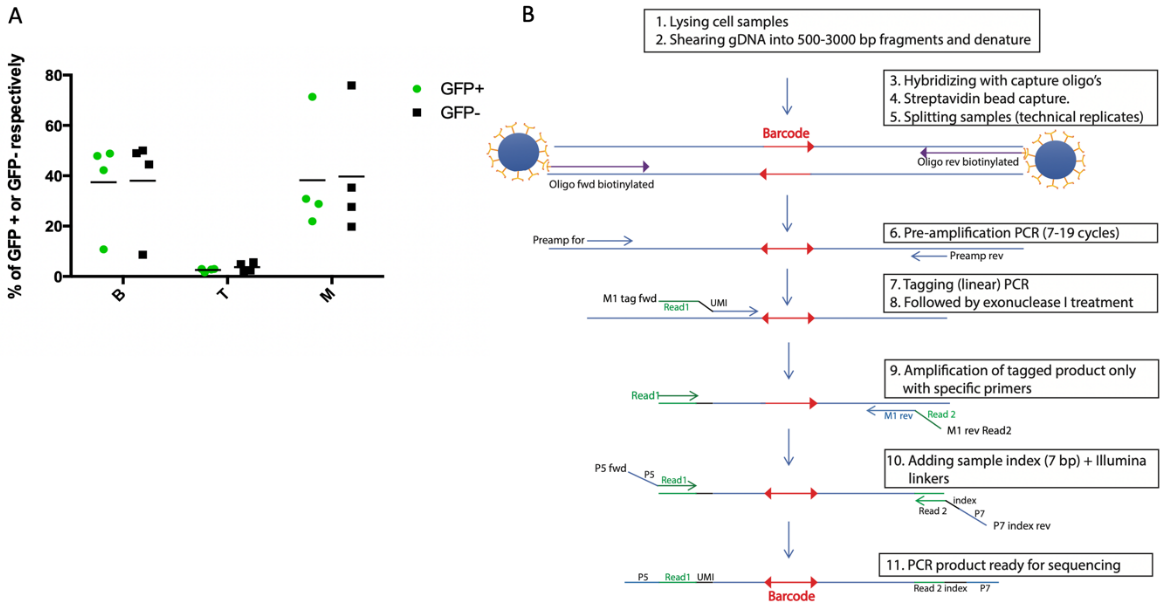

Figure S2: A. DRAG induction is neutral with respect to cell differentiation. Data depict the percentage of B cells ($CD19^+$), T cells ($CD3^+$) and myeloid cells ($CD11b^+$) within either the GFP^+ (green) or GFP^- (black) cell population in blood of tamoxifen-induced mice, 9 months after induction. The line represents the median and individual points the 4 mice analyzed. B. Sample processing pipeline for DRAG barcode amplification and deep sequencing.

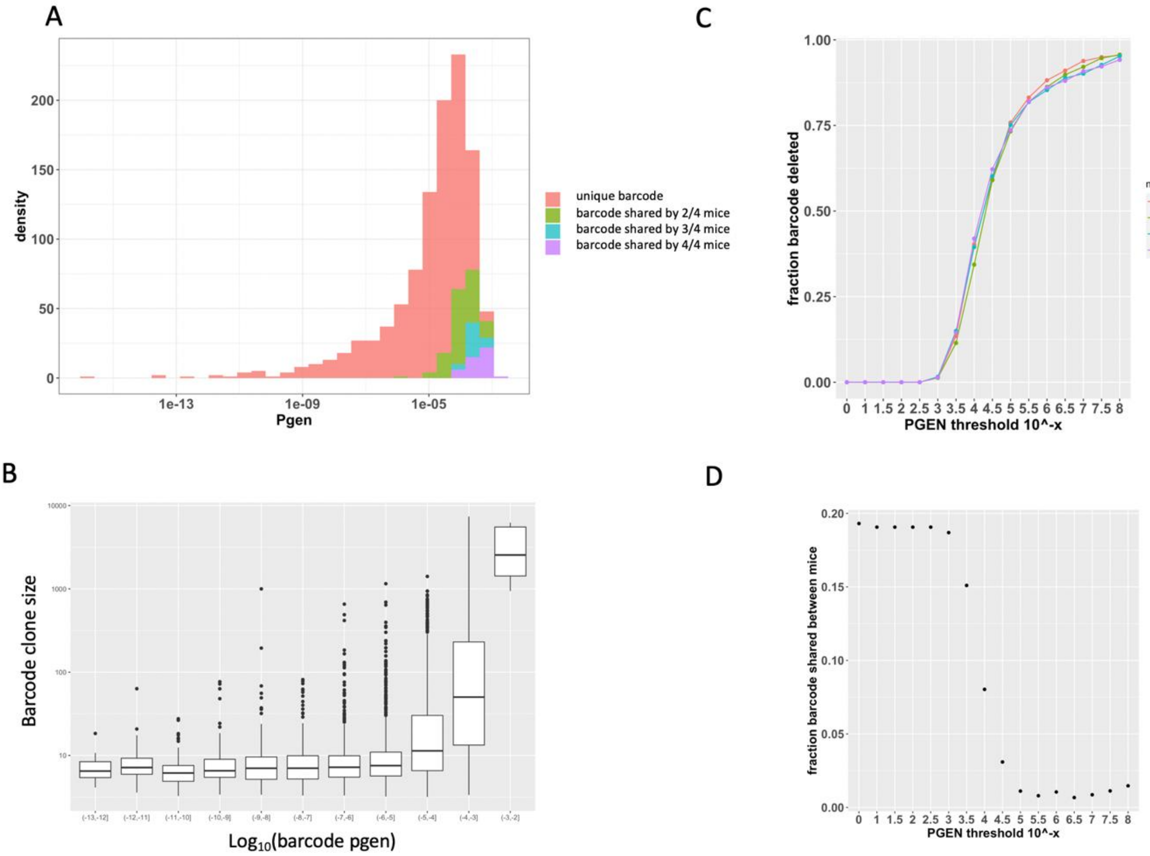

Figure S3: Quality control data for DRAG barcode recombination A. Distribution of the generation probability of barcodes (Pgen) of barcodes observed in an experiment using 4 mice. Pgen was calculated using the model described in the methods section. B. Frequency of reads of barcodes as a function of their estimated Pgen. N = 4 mice C. Fraction of total barcodes discarded per mouse for different Pgen threshold values (Pgen < 10^{-x}). For example, when using Pgen<10^{-4}, on average 39+/-3% of barcodes are discarded. N = 4 mice. The boxplot represents the median and interquartile range. The whiskers extend to 1.5 times the interquartile range values. D. Fraction of barcodes that are present in more than one mouse for different values of the Pgen threshold (Pgen < 10^{-x}). For example, when using Pgen<10^{-4}, 92% of the retained barcodes were unique to an individual mouse. N = 4 mice.

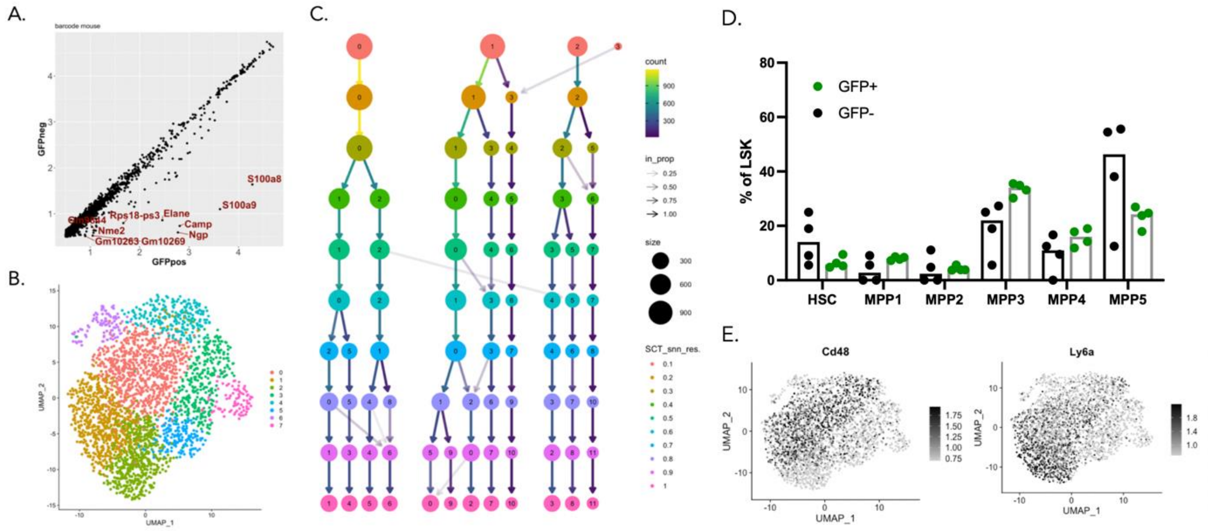

Figure S4: Supplementary Data for scRNAseq analysis in Fig 2. A. Comparison of the mean log expression of genes between GFP^{+} and GFP^{-} LSK cells prior to batch correction. B. Unsupervised Louvain clustering analysis of the LSK compartment using the Seurat package plotted using a UMAP representation. C. Cluster stability analysis for scRNAseq profiling of HSPCs, showing the relationship between clusters at different resolution parameters used in Seurat. The size of each node represents the number of cells in each cluster and colors represent different values of the resolution parameter in Seurats' implementation of the Louvain clustering algorithm. D. Flow cytometry quantification of proportion of GFP+ and GFP- HSPC subsets of 6 month old mice. Each point represents 1 mouse and n = 4. No statistically significant differences between GFP- and GFP+ representation amongst the HSPC subsets were observed. Statistical significance was tested using a paired two-sided Wilcoxon-Test. Full gating strategy is provided in Fig 7A. E. Overlay of *Cd48* and *Ly6a* expression onto the UMAP representation of the data. All data are from 2 mice induced at 20 weeks.

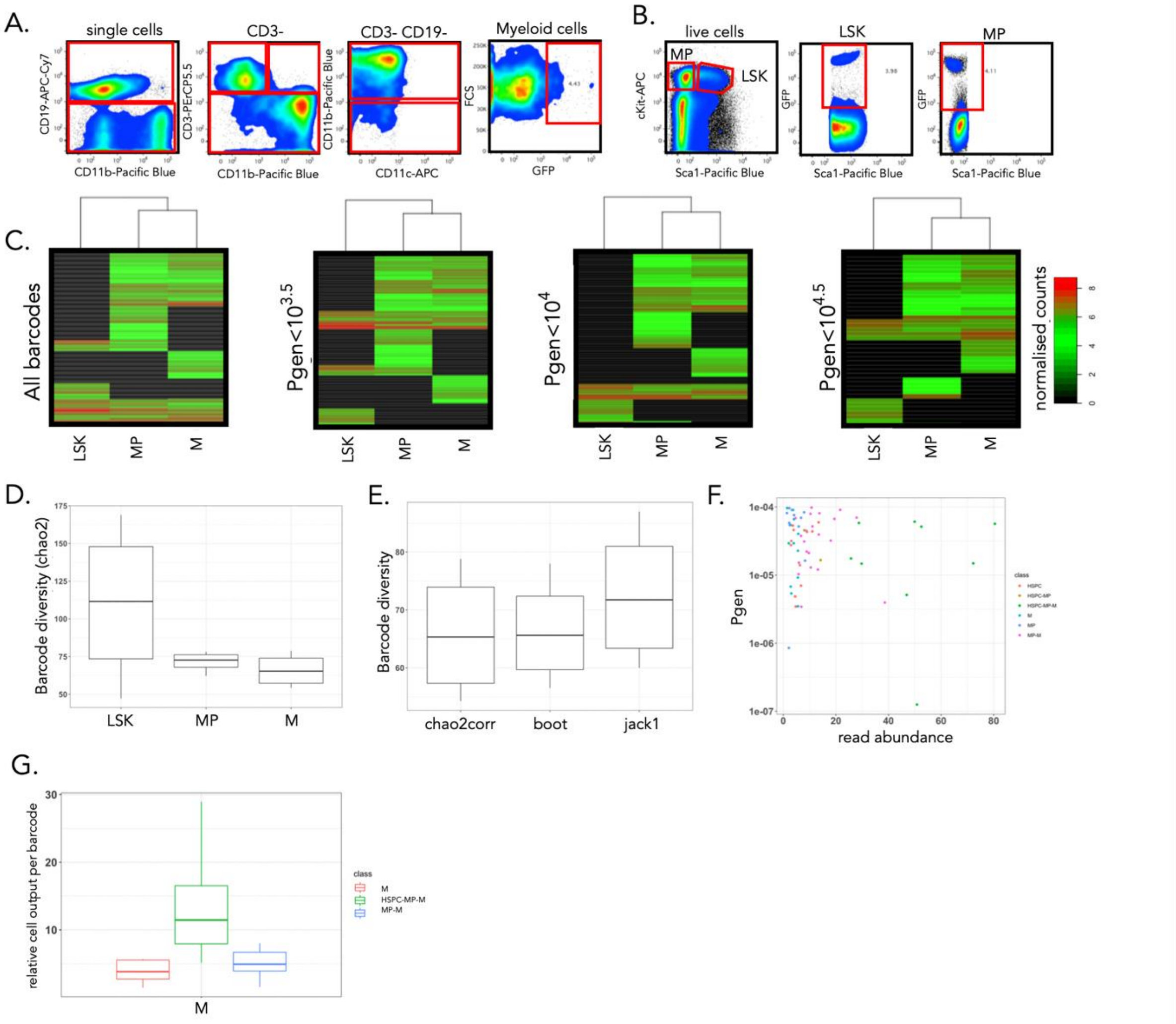

Figure S5: Supporting analyses for Fig 3. A. Cell sorting strategy used to obtain bone marrow myeloid cells (c-Kit enriched fraction) and blood myeloid cells. In addition, a representative plot of GFP expression in the sorted cell population is depicted. B. Cell sorting strategy for bone marrow myeloid progenitors (MP) and HSPC (LSK) from the c-Kit enriched fractions of whole bone marrow. C. Heatmap representation of barcode output in bone marrow HSPC, MP, and myeloid cells, as defined in Fig3B, at month 15 post-induction. Data are depicted for different values of the threshold for barcode generation probability Pgen (retaining 227, 164, 97, or 42 barcodes for analysis for the Pgen values depicted from left to right). Pooled data of 4 mice, renormalized, arcsine transformed data clustered by complete linkage using Euclidean distance are depicted. D. Chao2 estimate of the diversity of barcodes in bone marrow HSPC, MP, and myeloid cells in bone marrow and blood 15 months post-induction. mean and SD over 4 mice. The boxplot represents the median and interquartile range. The whiskers extend to 1.5 times the interquartile range values. E. Comparison of different diversity estimators based on abundance data. The estimators used are the bias-corrected chao2 (chao2corr), the first order jackknife (jack1), the bootstrap (boot). The boxplot represents the median and interquartile range. The whiskers extend to 1.5 times the interquartile range values. F. Probability of DRAG barcode generation

as a function of read abundance, color coded by the different classes of output as in Fig 3C and 3D. G. The relative cell output represents the fraction of reads per barcode of the total reads found in myeloid cells. This relative cell output is presented per barcode category as defined in Fig 3C and 3D. The boxplot represents the median and interquartile range. The whiskers extend to 1.5 times the interquartile range values. The mean and SD over barcodes obtained from 4 mice is displayedN = 4 mice. The colors represent the barcode categories as in Fig 3C and 3D.

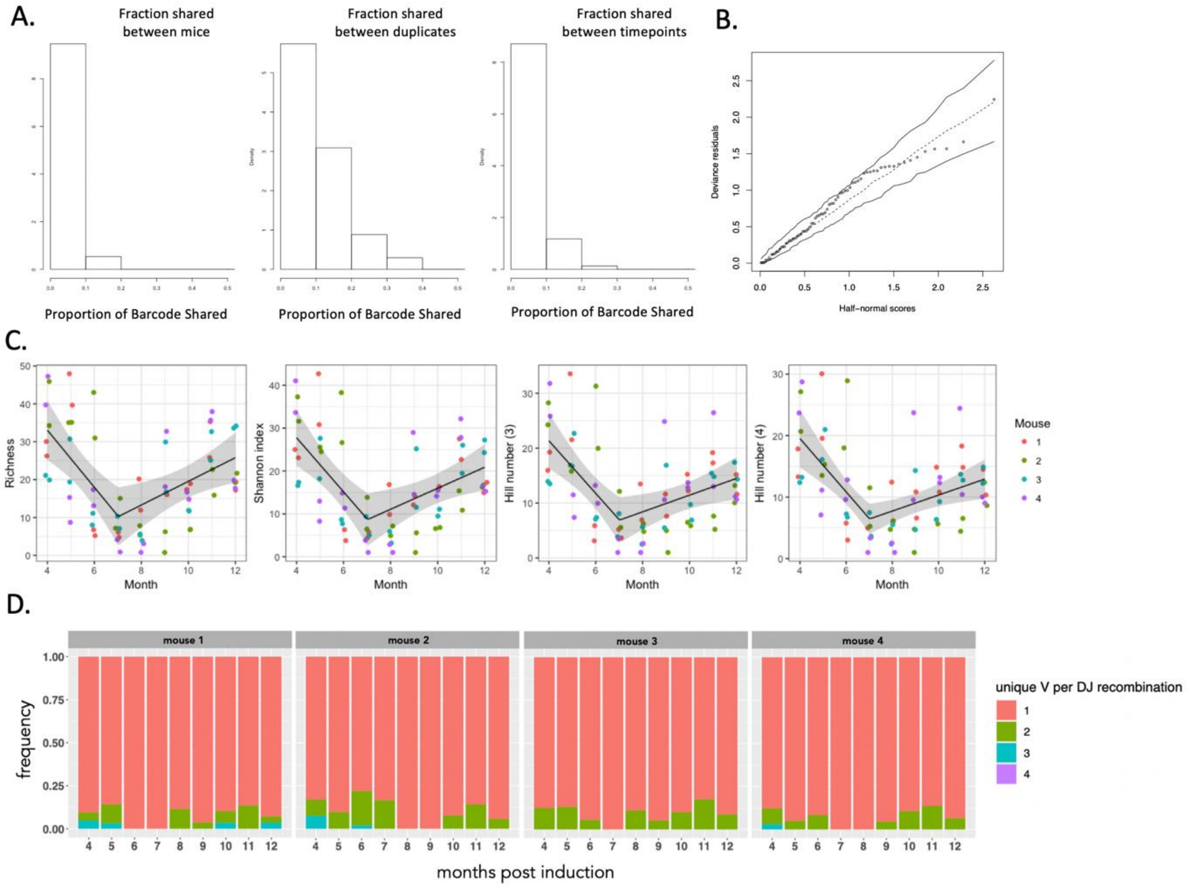

Figure S6: Supporting analyses for Fig 3. A. Fraction of barcodes in myeloid cells shared between mice (indicative of frequently occurring barcodes), between duplicates (indicative of sampling efficiency at a given time point) and between time points. Pooled data of four mice for the experimental set up shown in Fig 3A. B. Half-normal plot of the conditional deviance residuals for the generalized linear mixed model (GLMM) used in Fig 3G including a simulated envelope. The simulated envelope (solid lines) is obtained by simulating 99 response variables assuming the fitted model is the true model, refitting the same GLMM to the simulated samples, then obtaining and sorting the conditional deviance residuals in absolute value, and calculating the 2.5% and 97.5% percentiles for each order statistic, while the dashed line represents the median. The envelope is such that for a well-fitted model, most points are expected to fall within the envelope. In this case, all 72 points lie within the simulated envelope. C. Four different diversity estimates (Richness, Shannon index, Hill 3 and 4 number) of the barcodes in the myeloid cells in blood between 4 months and 12 months per mouse. Each sample was measured in

duplicate. The black line is the best fitted value of the gamma generalized linear mixed model with a break point. The grey ribbon represents the 95% CI for the true means. D. For each D and J recombination, the number of associated V regions was computed across all barcodes. The % of total barcodes (recombination) that had one, two or more V regions associated with one DJ recombination is plotted for the indicated times after barcode induction. The color represents the number of associated V regions and each of the four graph displays the result from one of four mice.

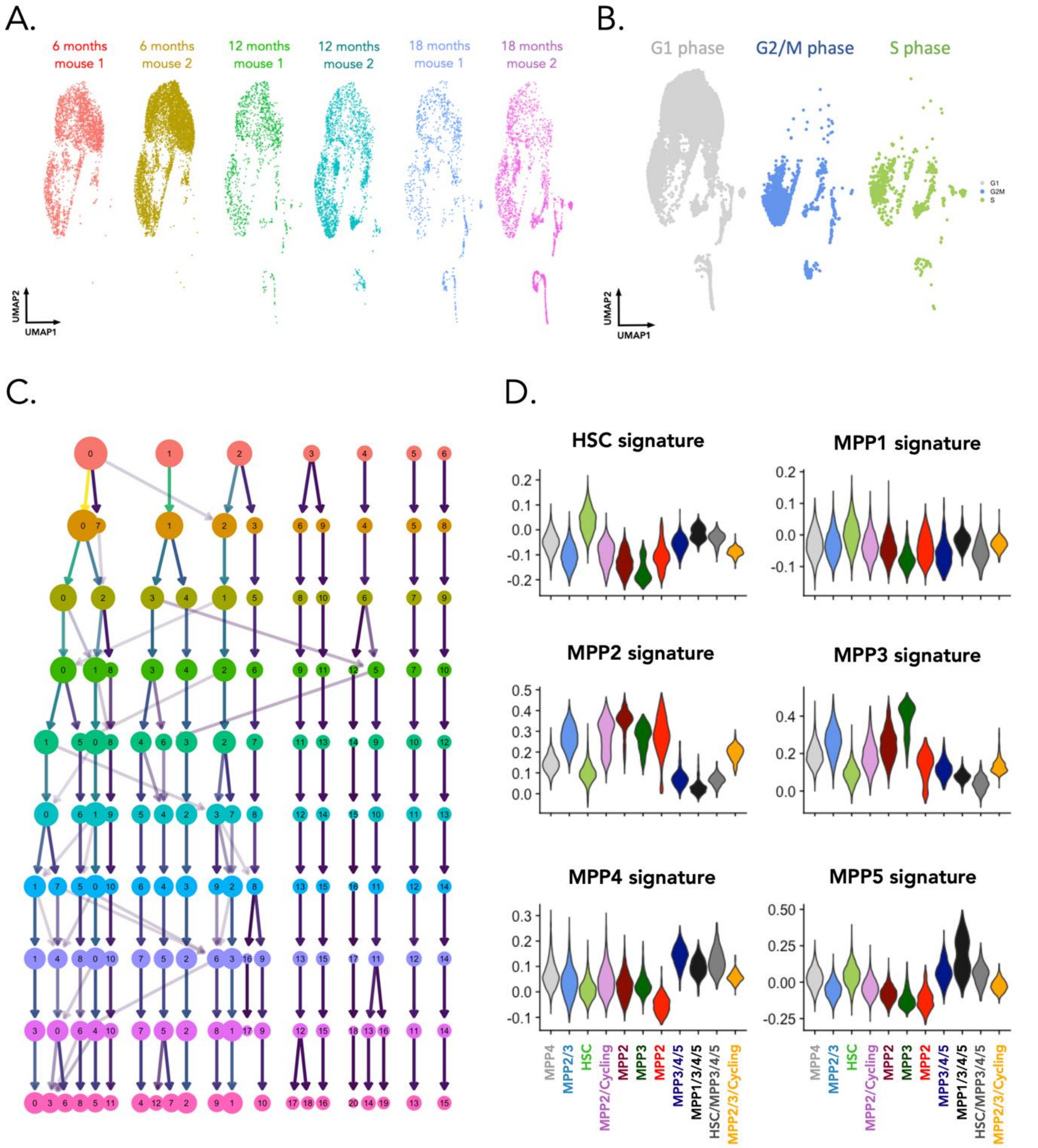

Figure S7: Supporting analyses for scRNAseq profiling of aged HSPCs (A) Cells from each individual mouse overlaid onto the UMAP embedding of the integrated data. (B) Cells from different cell cycle stages overlaid onto the UMAP embedding of the integrated data. Cell cycle stage was annotated using the classifier-based approach from [28] implemented as the cyclone method in the scran R package.(C) Cluster stability analysis. Each row represents a different

resolution parameter of the Seurat default clustering algorithm. Each node represents a cluster and arrows represent the relationship between clusters across different resolution parameters. The size of each node is scaled to the number of cells in the respective cluster. (D) Expression of HSC and MPP signatures from Sommerkamp et al (2021) amongst different clusters. Signature expression for each cell was calculated by taking the mean expression across all genes (after background correction)

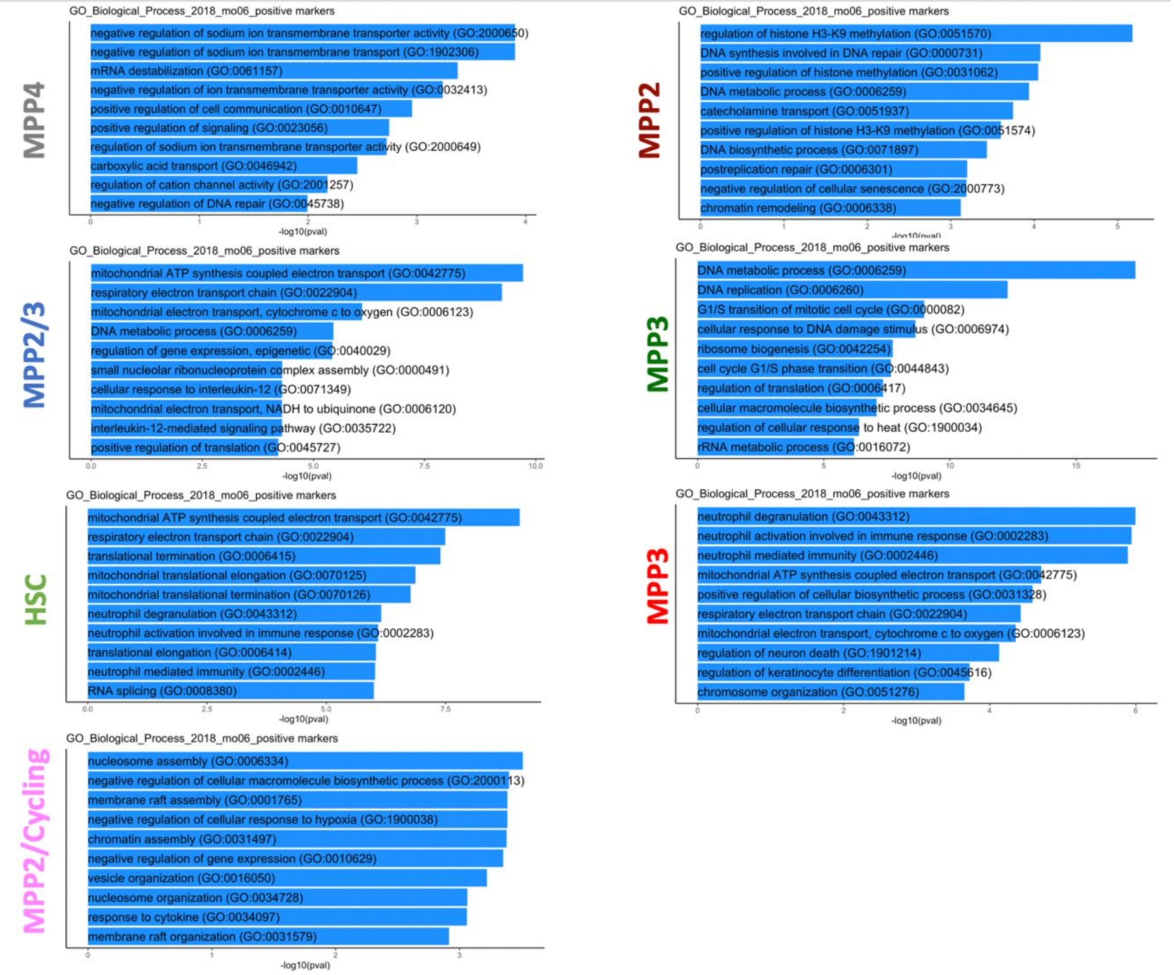

Figure S8: Pathways upregulated in HSPCs from aged mice across each cluster. Pathways enriched in HSPC clusters from mice aged 19 months compared to equivalent HSPC clusters from mice aged 6.5 months. Some clusters did not contain cells from 6.5 month old mice and were hence excluded from this analysis. Pathway analysis was performed using the enrichR R package using a variation of Fisher's exact test (two-sided), which also considers the size of each gene set when assessing its statistical significance.

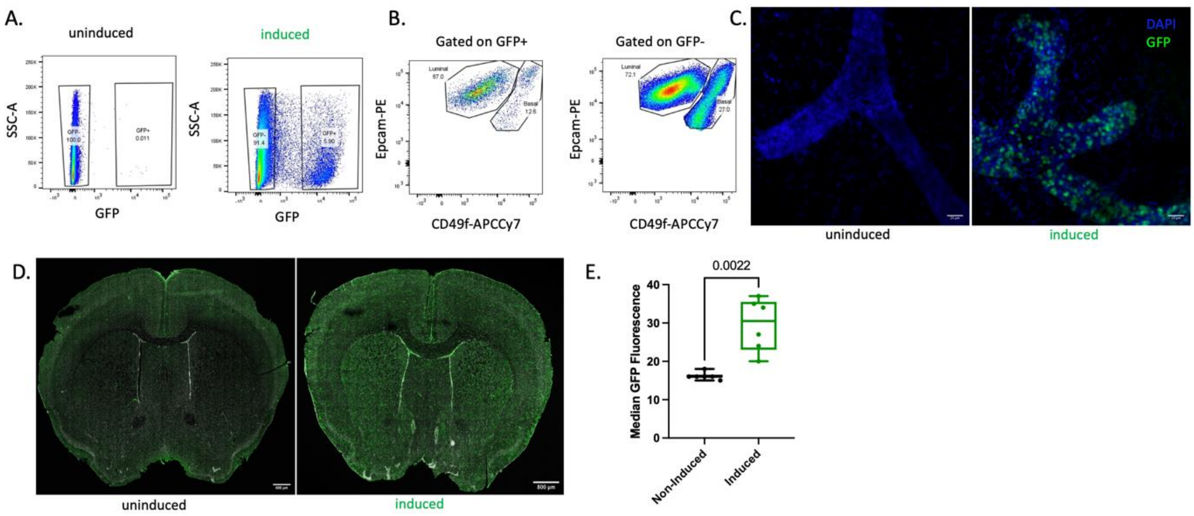

Figure S9: DRAG barcoding in the mammary gland and the brain. (A) Representative GFP+ cell population in mammary epithelial cells of an uninduced mouse (left) and induced mouse (right) 1 month post-induction. (B) Representative flow cytometry dot plots of luminal and basal cells gated within the mammary epithelial GFP positive (left) and GFP negative (right) populations. (C) Maximum projection of whole mount mammary gland from uninduced and induced DRAG mice 1 month post-induction, showing DAPI and GFP signal. Example data from 1 mouse and 1 experiment (D) Exemplary images of GFP^+ signal from barcoded cells in brain tissue sections from uninduced and induced DRAG mice and (E) Quantification of GFP fluorescence intensities in uninduced vs induced DRAG mice from different sections of the brain. Each point represents a different tissue section from 2 induced and 2 uninduced mice (3 tissue sections per mouse). Statistical comparisons were made using a two-sided Mann-Whitney test. Boxplots represent the median and IQR, whiskers extend to the min and max values.

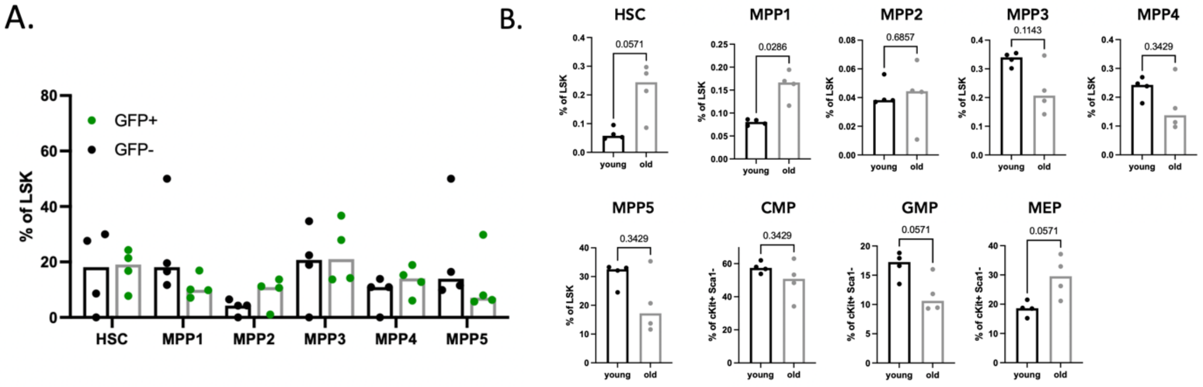

Figure S10. Flow cytometry analysis of HSPC and MP subsets in young and old mice. A. Flow cytometric quantification of the proportion of GFP+ and GFP- HSPC subsets in mice aged 19 months. Each point represents 1 mouse and n = 4. No statistically significant differences between GFP- and GFP+ representation amongst the HSPC subsets were observed. Statistical significance was tested using a paired two-sided Wilcoxon-Test. B. Quantification of cKit+ Sca1+ HSPCs and cKit- Sca1- MP subset frequencies between young (6.5 months) and old (19 months) mice. Each point represents 1 mouse and n = 4 mice. Statistical comparisons were made using a two-sided Mann-Whitney test.

Supplementary references

1. Altschul, S. F., Gish, W., Miller, W., Myers, E. W. & Lipman, D. J. Basic local alignment search tool. *J Mol Biol* (1990) doi:10.1016/S0022-2836(05)80360-2.
2. Marcou, Q., Mora, T. & Walczak, A. M. High-throughput immune repertoire analysis with IGoR. *Nat Commun* (2018) doi:10.1038/s41467-018-02832-w.
3. R Core Team. R: A language and environment for statistical computing. R Foundation for Statistical Computing. (2019).
4. Oksanen, J. *et al.* vegan: Community Ecology Package. (2018).
5. Jost, L. Entropy and diversity. *Oikos* Preprint at https://doi.org/10.1111/j.2006.0030-1299.14714.x (2006).
6. Warnes, G. R. *et al.* Package 'gplots': Various R programming tools for plotting data. *R package version 2.17.0.* (2016) doi:10.1111/j.0022-3646.1997.00569.x.
7. Naik, S. H. *et al.* Diverse and heritable lineage imprinting of early haematopoietic progenitors. *Nature* **496**, (2013).

8. Perié, L., Duffy, K. R., Kok, L., de Boer, R. J. & Schumacher, T. N. The Branching Point in Erythro-Myeloid Differentiation. *Cell* **163**, (2015).
9. Thuiller, W., Georges, D., Engler, R. & Breiner, F. biomod2: Ensemble Platform for Species Distribution Modeling. (2016).
10. Dray, S. & Dufour, A. B. The ade4 package: Implementing the duality diagram for ecologists. *J Stat Softw* (2007) doi:10.18637/jss.v022.i04.
11. Wei, T. & Simko, V. (2017). R. package 'corrplot': V. of a C. M. (Version 0. 84). A. from https://github. com/taiyun/corrplot. R package 'corrplot': Visualization of a Correlation Matrix. (2017).
12. Moral, R. A., Hinde, J. & Demétrio, C. G. B. Half-normal plots and overdispersed models in R: The hnp package. *J Stat Softw* (2017) doi:10.18637/jss.v081.i10.
13. Bates, D. M., Maechler, M., Bolker, B. & Walker, S. lme4: linear mixed-effects models using S4 classes. *J Stat Softw* (2015).
14. Brooks, M. E. *et al.* glmmTMB balances speed and flexibility among packages for zero-inflated generalized linear mixed modeling. *R Journal* **9**, 378–400 (2017).
15. Hafemeister, C. & Satija, R. Normalization and variance stabilization of single-cell RNA-seq data using regularized negative binomial regression. *Genome Biol* (2019) doi:10.1186/s13059-019-1874-1.
16. McInnes, L., Healy, J., Saul, N. & Großberger, L. UMAP: Uniform Manifold Approximation and Projection. *J Open Source Softw* (2018) doi:10.21105/joss.00861.
17. Blondel, V. D., Guillaume, J. L., Lambiotte, R. & Lefebvre, E. Fast unfolding of communities in large networks. *Journal of Statistical Mechanics: Theory and Experiment* (2008) doi:10.1088/1742-5468/2008/10/P10008.
18. Wilson, N. K. K. *et al.* Combined Single-Cell Functional and Gene Expression Analysis Resolves Heterogeneity within Stem Cell Populations. *Cell Stem Cell* **16**, 712–724 (2015).
19. Pietras, E. M. *et al.* Functionally Distinct Subsets of Lineage-Biased Multipotent Progenitors Control Blood Production in Normal and Regenerative Conditions. *Cell Stem Cell* **17**, 35–46 (2015).
20. Macaulay, I. C. *et al.* Single-Cell RNA-Sequencing Reveals a Continuous Spectrum of Differentiation in Hematopoietic Cells. *Cell Rep* **14**, 966–977 (2016).
21. Nestorowa, S. *et al.* A single-cell resolution map of mouse hematopoietic stem and progenitor cell differentiation. *Blood* **128**, e20 (2016).
22. Pellin, D. *et al.* A comprehensive single cell transcriptional landscape of human hematopoietic progenitors. *Nature Communications 2019 10:1* **10**, 1–15 (2019).
23. Velten, L. *et al.* Human haematopoietic stem cell lineage commitment is a continuous process. *Nat Cell Biol* (2017) doi:10.1038/ncb3493.
24. Sommerkamp, P. *et al.* Mouse multipotent progenitor 5 cells are located at the interphase between hematopoietic stem and progenitor cells. *Blood* **137**, 3218–3224 (2021).
25. Kuleshov, M. V. *et al.* Enrichr: a comprehensive gene set enrichment analysis web server 2016 update. *Nucleic Acids Res* **44**, W90–W97 (2016).
26. Chen, E. Y. *et al.* Enrichr: Interactive and collaborative HTML5 gene list enrichment analysis tool. *BMC Bioinformatics* **14**, 1–14 (2013).
27. Wilson, N. K. *et al.* Combined Single-Cell Functional and Gene Expression Analysis Resolves Heterogeneity within Stem Cell Populations. *Cell Stem Cell* **16**, 712–724 (2015).
28. Scialdone, A. *et al.* Computational assignment of cell-cycle stage from single-cell transcriptome data. *Methods* **85**, 54–61 (2015).
