# Supplementary material for: DRAG in situ barcoding reveals an increased number of HSPCs contributing to myelopoiesis with age
Source: Nat Commun. 2023 Apr 17;14:2184. doi: 10.1038/s41467-023-37167-8 (PMC10110593; doi:10.1038/s41467-023-37167-8)
Supplement: Supplementary file 3 — Description of Additional Supplementary Files [file 41467_2023_37167_MOESM3_ESM.docx]

**Supplementary Data 1: List of i7 indexes**

**Supplementary Data 2: Comparisons between in situ barcoding methods**

**Supplementary Data 3: Differentially expressed genes across clusters (Fig 5).**

Differential gene expression analysis was performed using a two-sided logistic regression test as implemented in the Seurat R package and Bonferroni correction was applied to account for multiple testing.

**Supplementary Data 4: Differentially expressed genes across HSPCs taken from mice of different ages**

Differential gene expression analysis was performed using a two-sided logistic regression test as implemented in the Seurat R package and Bonferroni correction was applied to account for multiple testing.

**Supplementary Data 5: Pathway enrichment analysis comparing HSPCs taken from mice of different ages**

Pathway analysis was performed using the enrichR R package using a variation of Fisher’s exact test (two-sided), which also considers the size of each gene set when assessing the statistical significance of a gene set

**Supplementary Data 6: Proportion of sample/cell type abundances for stacked barplots**
