# Supplementary material for: DRAG in situ barcoding reveals an increased number of HSPCs contributing to myelopoiesis with age
Source: Nat Commun. 2023 Apr 17;14:2184. doi: 10.1038/s41467-023-37167-8 (PMC10110593; doi:10.1038/s41467-023-37167-8)
Supplement: Supplementary file 10 — Reporting Summary [file 41467_2023_37167_MOESM10_ESM.pdf]

nature portfolio

Corresponding author(s): Leila Perie

Last updated by author(s): Feb 6, 2023

Reporting Summary

Nature Portfolio wishes to improve the reproducibility of the work that we publish. This form provides structure for consistency and transparency in reporting. For further information on Nature Portfolio policies, see our Editorial Policies and the Editorial Policy Checklist.

Statistics

For all statistical analyses, confirm that the following items are present in the figure legend, table legend, main text, or Methods section.

n/a	Confirmed	
☐	☒	The exact sample size (*n*) for each experimental group/condition, given as a discrete number and unit of measurement
☐	☒	A statement on whether measurements were taken from distinct samples or whether the same sample was measured repeatedly
☐	☒	The statistical test(s) used AND whether they are one- or two-sided<br>*Only common tests should be described solely by name; describe more complex techniques in the Methods section.*
☐	☒	A description of all covariates tested
☐	☒	A description of any assumptions or corrections, such as tests of normality and adjustment for multiple comparisons
☐	☒	A full description of the statistical parameters including central tendency (e.g. means) or other basic estimates (e.g. regression coefficient) AND variation (e.g. standard deviation) or associated estimates of uncertainty (e.g. confidence intervals)
☐	☒	For null hypothesis testing, the test statistic (e.g. *F*, *t*, *r*) with confidence intervals, effect sizes, degrees of freedom and *P* value noted<br>*Give P values as exact values whenever suitable.*
☒	☐	For Bayesian analysis, information on the choice of priors and Markov chain Monte Carlo settings
☒	☐	For hierarchical and complex designs, identification of the appropriate level for tests and full reporting of outcomes
☐	☒	Estimates of effect sizes (e.g. Cohen's *d*, Pearson's *r*), indicating how they were calculated

Our web collection on statistics for biologists contains articles on many of the points above.

Software and code

Policy information about availability of computer code

Data collection	No software was used in the collection of data for this study
Data analysis	Data analysis for flow cytometry/FACS was performed using FlowJo v10.2 software (TreeStar) and Prism v9.<br>Barcoding demultiplexing was performed using Xcalibr (v1).<br>Barcoding analysis was performed using a custom bioinformatics pipeline implemented in the R programming language (v3-v4). The R packages we use are ggplot2 (3.3.6), ,glmmTMB (1.5.3), lme4 (1.1-29)<br>scRNAseq preprocessing was performed in CellRanger (v2.1.1 and v6) and analysis was performed in R using the Seurat (v3-v4),destiny (v3.10), and enrichr (v3) packages<br><br>All code is available at: https://github.com/TeamPerie/UrbanusCosgrove-et-al-DRAG-mouse

For manuscripts utilizing custom algorithms or software that are central to the research but not yet described in published literature, software must be made available to editors and reviewers. We strongly encourage code deposition in a community repository (e.g. GitHub). See the Nature Portfolio guidelines for submitting code & software for further information.

Data

Policy information about availability of data

All manuscripts must include a data availability statement. This statement should provide the following information, where applicable:

- Accession codes, unique identifiers, or web links for publicly available datasets
- A description of any restrictions on data availability
- For clinical datasets or third party data, please ensure that the statement adheres to our policy

Raw sequencing data has been deposited into the zenodo database in .fastq file format or as .tsv cellranger outputs. Links to download these data are provided in the source data file. Preprocessed data are available at https://github.com/TeamPerie/UrbanusCosgrove-et-al-DRAG-mouse.git along with source code to generate figures from these data. Source data for all other data types (e.g. flow cytometry) are provided in the supplementary information and the source data file.

Human research participants

Policy information about studies involving human research participants and Sex and Gender in Research.

Reporting on sex and gender	NA
Population characteristics	NA
Recruitment	NA
Ethics oversight	NA

Note that full information on the approval of the study protocol must also be provided in the manuscript.

Field-specific reporting

Please select the one below that is the best fit for your research. If you are not sure, read the appropriate sections before making your selection.

☒ Life sciences ☐ Behavioural & social sciences ☐ Ecological, evolutionary & environmental sciences

For a reference copy of the document with all sections, see nature.com/documents/nr-reporting-summary-flat.pdf

Life sciences study design

All studies must disclose on these points even when the disclosure is negative.

Sample size	For barcode/immunophenotyping studies we used n = 4 mice per experiment, this number was based on pilot studies that we had performed in the lab. For transcriptomics studies we used n = 2 mice per experiment to have a biological replicate at each timepoint
Data exclusions	For all sequencing experiments (lineage barcodes and scRNAseq) we perform an initial quality control of the data. Here data are filtered based on a number of criteria including the number of reads per cell/sample, and the consistency of read counts across technical replicates. A full detailing of our data filtering procedures are provided in the supplementary materials and methods. We confirm that on the basis of these criteria no samples were excluded from the study
Replication	The number of experimental replicates for each figure is provided in the appropriate figure legends.
Randomization	The major comparisons that we make in this study are between young and aged mice. In this setting we were not able to randomize based on cages.
Blinding	No blinding was incorporated into our study design. Due to the highly technical nature of the experiments we have performed the researchers performing the experiments also performed the data analysis.

Reporting for specific materials, systems and methods

We require information from authors about some types of materials, experimental systems and methods used in many studies. Here, indicate whether each material, system or method listed is relevant to your study. If you are not sure if a list item applies to your research, read the appropriate section before selecting a response.

Materials & experimental systems

n/a	Involved in the study
	☒ Antibodies
☒	☐ Eukaryotic cell lines
☒	☐ Palaeontology and archaeology
	☒ Animals and other organisms
☒	☐ Clinical data
☒	☐ Dual use research of concern

Methods

n/a	Involved in the study
☒	☐ ChIP-seq
	☒ Flow cytometry
☒	☐ MRI-based neuroimaging

Antibodies

Antibodies used

All antibodies used in the study are listed in the materials and methods under the respective sections. A full list of the antibodies used in this study is:
CD117 (c-kit APC, clone 2B8, Biolegend, cat number = 105812,dilution 1/100),
Sca-1 (APC-Cy7, clone D7, Biolegend, 1/100, cat number = 108126),
CD135 (Flt3 PE-Cy5, clone A2F10, Life technologies, 1/50, cat number = 15-1351-82),
CD150 (Slam Pecy7, clone TC15-12F12.2, Biolegend, 1/100, cat number = BLE115914),
CD48 (Pacific Blue, HM48-1, Biolegend, 1/100, cat number = 103417),
CD16/32 (PercPCy5.5, clone 2.4G2, BD Bioscience, 1/100, cat number = 560540),
CD34 (Alexa 700, 1/100),
Lineage cocktail (PE, CD3ε clone 145-2C11; Ly-6G/Ly-6C clone RB6-8C5; CD11b clone M1/70; CD45R/B220 clone RA3-6B2; TER-119, Biolegend, 1/200, cat number = 133303).
CD11b (PercPCy5.5, clone M1/70, ebioscience, 1/100, cat number = 480112-82),
Ly6C (APC, HK1.4, Thermofisher, 1/200, cat number = 17-5932-82),
Ly6G (BV510, RUO, Biolegend, 1/100, cat number = 127633),
Siglec F (PE CF594, RUO, BD 1/100, cat number = 562757),
F4/80 (alexa 700, clone BM8, Ozyme, 1/100, cat number = 123130),
B220 clone RA3-6B2 Biolegend, PeCy7, 1/100, cat number = 103232,
CD3, clone 17A2 Biolegend, PeCy7,1/100, cat number = 100220,
CD11c clone N418, ebioscience, PeCy7, 1/100, cat number = 25-0114-82,
NK1.1 clone PK136 Biolegend, PeCy7, 1/100, cat number = 108714,
Ter119 clone TER-119 BD Biosciences) PeCy7, 1/100, cat number = 557853,
Sca-1 (Pacific Blue, clone D7, eBioscience, 1/200, cat number = 122520),
CD135 (Flt3 PE, clone A2F10, ebiosciences, 1/100, cat number = 12-1351-83)
CD11C (APC, clone HC3, BD biosciences, 1/100, cat number = 17-0114-82)
CD19 (APC-Cy7, clone 1D3, BD Pharmingen, 1/100,cat number = 557655).
CD45 clone 30-F11, Biolegend, APC, 1/100, cat number = 103111,
CD31 clone MEC13.3, Biolegend, APC, 1/100, cat number = 102509,
Ter119+ clone TER-119, Biolegend, APC, 1/100 , cat number = 116211,
PE EpCAM (APC, Biolegend, clone G8.8, 1/100), cat number = 118205
CD49f (APC/Cy7 , Biolegend, clone GoH3, 1/100), cat number = 313627

Validation

All antibodies are standard antibodies used by us and many in the field. All antibodies were validated in our lab by flow cytometry where we compare staining profiles and cell yields against those reported in the literature (Perie et al 2015, Pietras et al 2015, Sommerkamp et al 2021, Challen et al 2021)

Animals and other research organisms

Policy information about studies involving animals; ARRIVE guidelines recommended for reporting animal research, and Sex and Gender in Research

Laboratory animals

CAGCre-ER+/- DRAG+/-
ROSACre-ERT2+/- DRAG+/-
All mice used in this study were adult males aged 12 weeks to 2 years old housed under a 12 hour light cycle under standard laboratory conditions with ambient temperature (18-26 degrees celsius) and humidity control (30-40%)

Wild animals

No wild mice were used in this study

Reporting on sex

In this study we use only male mice in our analyses. This is because the female were kept for breeding.

Field-collected samples

No field collected samples were used in this study

Ethics oversight

All animal breeding and experiments were performed in accordance with national guidelines and were approved by the Experimental Animal Committee of the NKI (DEC 09036) or Institut Curie (#16854-2018092412148925-v1).

Note that full information on the approval of the study protocol must also be provided in the manuscript.

Flow Cytometry

Plots

Confirm that:

- [x] The axis labels state the marker and fluorochrome used (e.g. CD4-FITC).
- [x] The axis scales are clearly visible. Include numbers along axes only for bottom left plot of group (a 'group' is an analysis of identical markers).
- [x] All plots are contour plots with outliers or pseudocolor plots.
- [x] A numerical value for number of cells or percentage (with statistics) is provided.

Methodology

Sample preparation

bone marrow: At sacrifice, BM was harvested from femurs, tibias and ilia and progenitor fractions were enriched using anti-CD117 magnetic beads (Miltenyi). The c-kit+/ckit- fractions were stained with antibodies against surface markers of interest (as defined in the antibodies section and in the materials and methods).
blood: 100-200 µl blood samples were directly harvested in 800 µl Erylysis buffer (80.2 g NH4Cl, 8.4g NaHCO3, 3.7 g disodium EDTA in 1 L H2O, pH 7.4), incubated on ice, diluted with 5 ml Erylysis buffer, washed with medium RPMI, resuspended in 0.5 ml 10% RPMI medium and put on ice overnight. Subsequently, blood cells were stained in 50 µl 2% FCS RPMI medium with antibodies against surface markers defined in the antibodies section and in the materials and methods.
Mammary gland: ROSACreERT2+/- DRAG+/- mice were induced by one single injection of tamoxifen (0.1mg/g body weight) at P21. Mammary glands were collected 1 month after induction. Single cell dissociation was performed through enzymatic digestion (5mg/ml collagenase (Roche, 57981821) and 200U/ml hyaluronidase (Sigma, H3884) for 1h30 at 37°C under agitation. Subsequently, cells were treated with trypsin for 1 min and DNAse I and dispase for 5min at 37°C. Cell suspension was filtered through a 40µm cell strainer, and cells were stained in FACS buffer (PBS, EDTA 5mM, BSA 1%, FBS 1%) using a 'lineage cocktail' in APC (CD45 clone 30-F11, CD31 clone MEC13.3, Ter119+ clone TER-119, all diluted 1/100), PE EpCAM (clone G8.8, 1/100), APC/Cy7 CD49f (clone GoH3, 1/100) and DAPI. All antibodies were purchased from Biolegend. Cells were analyzed on a FACSAriaTM flow cytometer (BD Biosciences), and results were analysed using FlowJo software.

Instrument

Cell sorting was performed on a FACSAriaTM (BD Biosciences) using a 70 um nozzle at precision 0/16/0 and high efficiency.

Software

Data analysis was performed using FlowJoTM v.10 (TreeStar). Data was then exported from FlowJo and imported in GraphPad Prism.

Cell population abundance

On the github (https://github.com/TeamPerie/UrbanusCosgrove-et-al-DRAG-mouse/tree/main/code%20for%20figures), there is a table called "cellnumbrun3and4" that provide the number of cells sorted for each population. The purity was checked for some samples in each experiments by re-aquiring the sorted samples. The gating strategy for our experiments is provided in figure 7A, S1B,S5A-B, S9A-B

Gating strategy

The gating strategy for our experiments is provided in figure 7A, S1B,S5A-B, S9A-B

- [x] Tick this box to confirm that a figure exemplifying the gating strategy is provided in the Supplementary Information.
